# Autonomous Farmers Use of Complementary and Alternative Veterinary Medicines in Pasture-Based Dairy Goat Systems

**DOI:** 10.3390/ani15111627

**Published:** 2025-05-31

**Authors:** Jacques Cabaret, Vincent Lictevout

**Affiliations:** 1 Institut National de la Recherche Agronomique et Environnement, Unité Mixte de Recherche 1282, 37380 Nouzilly, France; 2 SantéSocioVéto, 8 Place Carré de Busserolle, 37100 Tours, France; 3 Touraine Conseil Elevage, 38 Rue Augustin Fresnel, 37050 Chambray-les-Tours, France; vincent.lictevout@idele.fr

**Keywords:** farmer, autonomy, complementary medicine, dairy goat, pasture, veterinary advisory

## Abstract

Consumers expect ruminants to graze outdoors and flocks to be of a reasonable size. Dairy goats can be kept outdoors (natural) or indoors. There are few pasture-grazed-system goat farms. The farms we studied in the centre-west of France were either organic or conventional, and all used pastures and tended to meet consumer demand for naturalness. We obtained information through semi-directed interviews. Dairy goats are susceptible to gastrointestinal infections when using pastures and this was one of the main health problems mentioned by organic farmers. There are a very limited number of medicines available for lactating dairy goats and farmers can use complementary and alternative veterinary medicine (CAVM), where they are completely autonomous in their choice. The use of CAVM has also been driven by organic labels and protected designation of origin (PDO) regulations for goat cheese. Homeopathy, phytotherapy, and aromatherapy were used for various health problems on almost all the farms surveyed. Herd size and farm area were negatively associated with the complex use of CAVM, possibly due to the workload on larger farms. Some CAVMs were used more in relation to the management of farms: aromatherapy in organic farms and homeopathy in cheese-making farms. The farmers with higher levels of education were more likely to employ phytotherapy. The farmers were autonomous in the choice of CAVM and did not rely on the advice of veterinarians.

## Simple Summary

Dairy goats in France are either reared indoors on large farms or on pasture-based systems in smaller farms. Information on the latter was gathered through semi-directive interviews with organic and conventional dairy goat farmers in centre-west France. Due to the limited number of medicines available for dairy goats during lactation, farmers have largely turned to complementary and alternative veterinary medicine (CAVM). Homeopathy, phytotherapy, and aromatherapy were used for various health problems in nine of the ten farms surveyed. Herd size and farm area were negatively associated with the complex use of CAVM, possibly due to the workload on larger farms. Some CAVMs were more common in relation to farm management: aromatherapy in organic farms and homeopathy in cheese farms. Farmers with a higher level of education were more likely to use phytotherapy. Farmers were autonomous in their choice of CAVM and did not rely on the advice of veterinarians.

## 1. Introduction

While autonomy in veterinary care is debated, its impact on dairy goat health—particularly in pasture systems—remains understudied [1]. Unlike sheep [2,3,4], goat farmers’ reliance on CAVM lacks systematic documentation, despite its reported use in 16% of farms in Australia [5]. In sheep farming, under different conditions, it has been shown that animal health autonomy has not led to good results in the management of diseases like gastrointestinal nematode infections [2]. This lack of efficiency in parasite control was probably due to poor diagnosis combined with a lack of knowledge of complementary and alternative medicines. Autonomy is more developed in organic sheep farming than in conventional sheep farming. Conventional sheep farmers are mostly dependent on veterinarians, who provide them with synthetic drugs [3]. Some organic farms do not seem to manage the health strategy aspect easily and often spend more money on health care than their conventional counterparts [4]. The health strategy is therefore not entirely dependent on the type of production—conventional versus organic—but also on the farmer’s attitude towards life and nature [3]. The effectiveness of CAVM seems to be limited [6], although some argue the opposite (see Ref. [7] on homeopathy or Ref. [8] on phytotherapy). Complementary and alternative veterinary medicine refers to treatments, therapies, and/or modalities that are not accepted as part of mainstream veterinary practice but are used by some practitioners to treat animals [9]. Alternative practices include acupuncture, photomedicine (laser), herbal therapy (phytotherapy and aromatherapy), manual therapy (osteopathy), nutraceuticals and dietary supplements, and homeopathy [10], and they are documented in a widely known general veterinary medicine manual [11]. Complementary and alternative medicine is largely used in the human population [12,13]; therefore, it is not surprising that clients would request this type of medicine from their veterinarian for their animals [10]. Integrative medicine (including the use of complementary and alternative veterinary medicine) is taught in some form in veterinary colleges in the USA [10]. There has been no such teaching in Europe, but from 2019 a one-year course in phytotherapy has been available to French veterinarians [14]. Most dairy goats are found in Asia (52%) and Africa (35%), but the most organised market for goat milk is in Europe, particularly in France [15]. The European goat sector is specialised in milk production, mostly for industrial cheesemaking, while also supporting traditional on-farm production. Europe contributes 15% of the world’s total goat milk with only 5% of the goat population, due to high specialisation and commercialisation. This production is subsidised by the state from 4 to 8% of farm income. Goat husbandry has undergone several changes from 1960 to the present in France: (i) from marginal production using pastures in many areas (mountainous areas included), it concentrated into larger herds bred indoors in plain areas located in the centre-west and south-east regions of France [16]; (ii) the societal demand for local and natural products and globalisation of the economy has shaped dairy goats in large indoor breeding units, and alternatively, smaller units using pastures, with farmers often producing and selling their own cheese under signs of quality [17]. Modern dairy goat production is thus not limited to confinement operations, and in Europe and North America researchers and producers are revisiting pasture grazing to reduce costs, maintain natural behaviours, and enhance the environment [15]. The regulations for several quality cheeses like Sainte-Maure de Touraine, require that goats are Alpine, Saanen, or their crossbreed; a stocking rate of 10 goats/ha of area dedicated to hay, wrapping bales, or pasture; that fodder is at minimum 550 kg of dry matter per goat annually; and that concentrate feed is limited to a maximum of 330 kg/year/goat, and 75% of the food should be local [18]. These regulations are satisfying the societal demand for naturalness regarding animals and local production. The organic farming regulations include constraints on the use of synthetic chemicals for treatments and disinfection, among others: CAVMs can be used as an alternative. Farmers producing cheese and/or under organic regulations have constraints that meet the acceptability of husbandry [19]. Dairy goat farmers in Touraine (a subdivision of centre-west France) utilising pastures and either running organic farms and/or producing quality cheese are then an interesting population for studying the use of CAVM in a socially accepted type of husbandry. They were interviewed using the same semi-directive format as already used in other contexts for agricultural farms [20] or goat meat farms [21]. We hypothesise that farmer autonomy in CAVM use is shaped by farm type (organic/conventional), production goals (cheese/milk), and societal demands. This study examines these dynamics in Touraine’s pasture-based systems.

## 2. Material and Methods

**Figure 1:**
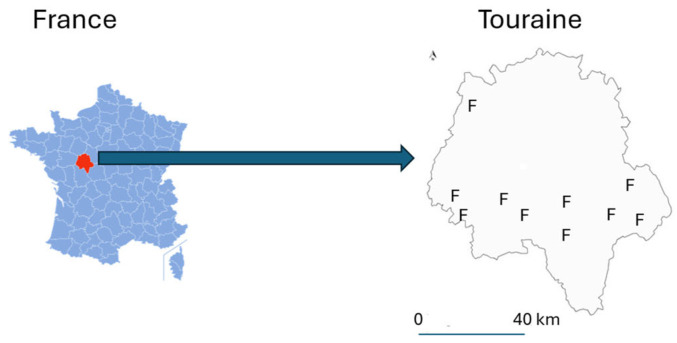
Location of the surveyed farms (F) in the centre-west of France.

Semi-directive interviews were conducted with 10 dairy goat farmers in Touraine (centre-west France) (Figure 1).

They were selected from a list of farmers who used pastures and who had been involved in goat rearing for at least 10 years. The use of pasture for dairy goats is a minority in Touraine (12% of the farms) [22]. The interviews were preceded by a short visit to the farm. The interviews were carried out with a guide of open questions and according to the life story method [23]. A life story is the story that the farmer chooses to tell about the life they have lived and what the teller wants others to know as a result of a guided interview by a researcher. Sociologists use life history to understand social reality (the use of CAVM) existing outside the story but described by the story. The interviews lasted for one hour or longer. Briefly, questions were asked about their personal and professional backgrounds, farm management and work, participation in collective activities, animal health and treatments (alternative or not) used, how they gained experience in animal health, and finally how they saw the future of their profession (Supplementary Dataset S1). The purpose of the farmer interviews was not to be representative, but to reconstruct the universe we are working with. The aim was to differentiate what brought them together or set them apart. This inclusive approach [24] was based on the farmers’ own experience and does not constitute a common frame of reference. The number of interviews usually varies between 10 and 30, depending on the saturation effect: they are stopped when they no longer provide new information [25], and for us 10 were sufficient. The recorded interviews were anonymised and transcribed into Word text. The Tropes (V8.5) [26] language analysis software was first used to process the data for the cognitive analysis of the interview [27], which was then analysed by multivariate methods [28] applied to the most frequently used words in the interview. Significant differences in the occurrence of words between farming types were assessed using z-score statistics for two populations; where proportions were low (less than 4%), Fisher’s exact test was applied to the number of occurrences of each word. Cluster analysis based on centroid grouping and Gower distance (since variables were binary, multistate, or quantitative) was performed using the MVSP software (version 3.1) [29]. A cluster analysis allowed all variables to be related simultaneously. The interviews were also classically analysed based on extracts of noteworthy sentences, mainly concerning care and alternative treatment practices. Each quotation is enclosed in quotation marks, with the reference to the farm (F1 to F10).

## 3. Results

### 3.1. Description of Farms (Table 1)

Nine out of the ten owners were men or couples. Eight farmers out of the ten had completed a two-year course in animal production after the ‘Baccalauréat’ (equivalent to a British A-level or US high school diploma). The farms ranged in size from 18 to 160 hectares and the herds from 80 to 400 goats. The main breeds were Alpine or Saanen or their crosses. They were dehorned or not, and on some farms small-ear phenotypes were recorded. The working units ranged from 1 to 10. Six farms prepared cheese and sold it on the farm and/or at the local market. Three farms were organic, and the others were conventional. All farms used CAVM (complementary and alternative medicine), but also conventional medicine.

**Figure 2:**
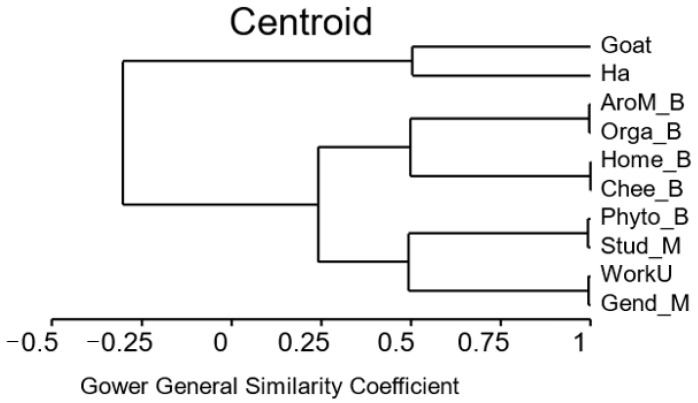
Cluster analysis (based on centroid) of the relationship between the characteristics of farms and use of complementary and alternative medicine: Quantitative, binary (B), multistate (M), size of the herd (Goat), surface area of the farm in hectares (Ha), working units (WorkU), gender of owners (Gend_M: 1: male, 2: female, 3: couple or more), level of studies (Stud_M: 1: below general certificate of education/A-level, 2: second-year university level in husbandry, 3: over this level), cheese making (Chee_B; yes or no), organic farming (Orga_B; yes or no), phytotherapy (Phyto_B; yes or no), aromatherapy (AroM_B; yes or no), homeopathy (Home_B; yes or no).

The size of the herd and area of the farm together are negatively related with the use of CAVM such as aromatherapy, phytotherapy, or homeopathy (Figure 2), possibly due to the workload on large farms. Aromatherapy use is related to organic farms (due to better confidence in CAVM), homeopathy with cheese making (absence of residues in the milk), and phytotherapy with the farmer’s level of studies (possibly due to a larger curiosity for exploring new practices).

### 3.2. Information from Word Occurrences for Dairy Goat Farms

#### 3.2.1. All Dairy Goat Farms

The occurrences of words are shown in Table 2. The idea of temporality is the most present. It means that the years are different and secondly that the time for work is short. The words work, milk, and treatment occur frequently and indicate the main concerns of the farmers. The word “organic” is mentioned because most of the farmers have an organic farming organisation or are interested in natural production. Veterinarians play a role in disease management and farmers use a variety of medicines obtained from them, often after having used CAVM themselves. The latter were divided into phytotherapy (plants, nutraceuticals, and food supplements based on plants), aromatherapy (from plants, but clearly separated by the farmers from other plant materials), homeopathy, the use of clay and, for one farmer, osteopathy. One farmer also practised magnetic healing.

#### 3.2.2. Differences Between Organic and Conventional Farms

**Figure 3:**
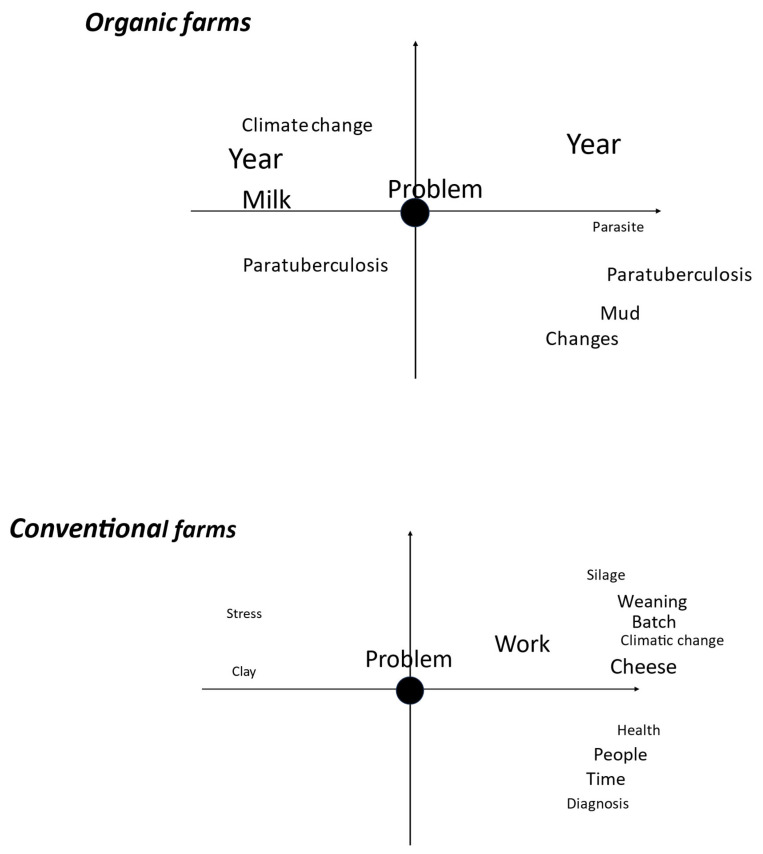
Qualitative content analyses of interviews of farmers practising dairy goat organic or conventional husbandry using Tropes software (version 3.1). The analyses are centred on the word problem; the left part of the figure corresponds to the words appearing before the central word and the right part to the words appearing after the central word; the distance between the central word and other words corresponds to the intensity of the relationship, strong if near, weak when distant. The size of font corresponds to the frequency of the word.

There was a significant difference (Fisher’s exact test, *p* = 0.001) in the occurrence of the words disease and phytotherapy, which were more common among organic farmers. The use of Tropes for the recorded interviews allowed a multidimensional analysis, which is presented in Figure 3. Organic and conventional farms share a concern about climate change, probably due to the use of pastures and cereals, the productivity of which depends on increasing drought in the temperate climate of France. Otherwise, they showed a number of differences. Organic farmers were most concerned about differences between years and milk production. Secondary problems were paratuberculosis, endoparasites, mud in the pastures, and the need for changes to adapt to the regulations. Conventional farmers had a wider range of minor problems, with workload and cheese making being the main concerns. Other concerns were the organisation of goat batches, weaning, and the use of silage. Health and diagnosis were a concern, and they were also aware of the importance of relationships with other people outside the farm. The word “silage” is probably related to the limited quantity authorised for the farmers by the regulation on local cheeses in Sainte-Maure de Touraine.

### 3.3. Remarkable Sentences of Farmers

#### 3.3.1. Role of Specifications Providing for Labels

The specifications are either organic (6 out of 10 farms) and/or Sainte-Maure de Touraine cheese (4/10) PDO (protected designation of origin). They are prescriptive and have a list of what to do or not: “We are under control; we do not have choice when we are producing cheese” (F3). The organic regulations may be considered as a guide. The organic label is also a way to differentiate yourself from others (F4). The farmers can also join Groups of Sanitary Protection (GDS: ‘Groupement de Défense Sanitaire’ in French), who provide services in the domain of health (8/10). A milk control service is also optional, undertaking periodic analyses of milk (4/10). These two organisations are not prescriptive, they identify health or production problems. These organisations, prescriptive or not, are rather considered as a guide for farmers: “I consider the organic regulations as a guide” (F9); “To be a member of Sainte Maure PDO, it helps, and with GDS, milk is tested once a week, *Staphylococcus* and *Listeria*, and also cells number in the milk, even if it is not such an accurate indicator of milk sanitary quality” (F7).

#### 3.3.2. Is CAVM Good for Animals?

Veterinarians in private practice are not seen frequently: “We see them for the compulsory annual veterinary visit of the herd and also for echography at the kidding period” (F6); “We do not call frequently the veterinarians, they deliver us with drugs for the goats on demand” (F4). They solely deliver drugs (synthetic allopathic molecules) with recognised efficacy against diseases. The farmers, organic or not, tend to solve sanitary problems by themselves: “If you have a sick goat, you will try plenty of things before using antibiotics, such as plants derived products… if it does not work, we will then ask for veterinary products” (F3); “We try a bit of everything before changing to veterinary products” (F4). They may wait until several unsolved cases appear before consulting a veterinarian (F7). One of the reasons is the cost of a veterinary visit: “The cost of veterinarian is high, but the cost of a goat is low…” (F9). Another reason is that they use human complementary and alternative medicine for themselves and believe in its efficacy: “We use homeopathy for ourselves. It corresponds to our ideas on terrain and equilibrium in diseases” (F6).

The opinions of farmers on CAVM are based on values and beliefs (plants and other CAVMs are natural so they should be good for the goat): “I use homeopathy and phytotherapy, I also bring them a tree so they can eat the bark. Magnetism (hand touch) is also employed- as the first CAVM in the herd. The most important is to believe in it…” (F5). They are also based on pragmatism (it has worked or not on several occasions): “We treat kids against coccidia. I tried vinegar, homeopathy and finally Vecoxan” (Vecoxan -MSD, Beaucouzé, France- is also known as diclazuril, and is a conventional coccidiocide) (F9). If satisfied, they write the recipe of the CAVM on a notebook to use it again., France

Their positive opinions are sometimes related to the acceptance of the product by the goat: “We give clay to the kids, and they love it”; possibly with the idea of goat self-medication: “Goats like brambles, blackthorn, bark, especially from oak” (F8).

#### 3.3.3. CAVM: Coping with Societal Demand

Agribashing is a sign that husbandry is not always appreciated: “The agribashing is due to the fact that people do not know husbandry, the citizen should be informed how animals are bred”. (F5). It can even frighten farmers: “It is a preoccupation, some set fire to the buildings at night. People are reticent, the industrial husbandry is not on top and that is why they point us”. (F2). The farmers noticed that “…consumers are more and more present. They are very demanding” (F1). They try to satisfy their clients by showing that they do not practise industrial farming: “The aim is to present a favourable image… by having a not too large herd”. (F5). Along the same lines, several farmers do not dehorn their goats: “We wanted goats with horns, because it is natural. When we were children, we saw goats with horns”. (F6). The farmers are not afraid of transparency: “Our farm is open. When clients have questions, we answer”. They also explain when they have problems with cheese production: “At the beginning we had *Pseudomonas* that renders the cheese bitter. We told the clients, we explained why”. (F6).

Another difficulty is the burden of climatic change, which is set to increase: “We have problem with mud when it rains. Seasons will change; we will have to reconsider our plannings. And during summer, we do not get water. We will have to change our habits”. (F1).

## 4. Discussion

There is an ongoing demand for effective and straightforward strategies for evaluating speech content analysis studies. Elo et al. [30] concluded that it is important to scrutinise the trustworthiness of every phase of the analysis process, including the preparation, organisation, and reporting of results. It is frequently difficult to obtain a representative sample. Our sample focused on dairy goat farmers utilising pastures, a less prevalent goat husbandry practice in the region of study. These farmers produced milk for dairy plants or prepared and sold cheese, predominantly Sainte-Maure PDO. Their farms were modest in size, with between 80 and 400 dairy goats. Representing approximately 12% of the dairy goat farms in the area [22], they offer a sustainable production model that may be more acceptable to consumers [19].The farms were selected by one of us (V.L.) as a first step in going beyond the usual scientific practice by (a) considering ecological and socioeconomic processes within the agricultural socio-ecosystem and (b) really involving stakeholdersthe research process to foster agroecological transitions [31]. 

The use of CAVM has been documented in organic cattle farms [32], among others, but only recently in conventional husbandry [5]. Dairy goat farmers use the different CAVM according to their beliefs that a more natural therapeutic is needed, because they can obtain them easily [33], and they do not require a milk production withdrawal period [34], and several have followed courses on these medicines (homeopathy, phytotherapy, and aromatherapy) to strengthen their autonomy. The shift towards more natural therapeutic solutions is influenced by concerns over the reliability of industrial farming methods and the use of synthetic chemicals. This shift is supported by initiatives such as the speed-dating event organised by Berkes et al. [35], which aims to facilitate direct interaction between farmers and citizens. However, the lack of competence in prescribing and administering medications has been identified as a key challenge in disease control, as evidenced by the study on parasites [36]. The efficacy of CAVM remains a subject of debate [9], yet phytotherapy has gained traction among farmers, with aromatherapy being less commonly employed. Aromatherapy was particularly favoured among farmers with a high level of education (see Figure 1). Homeopathy, when used (on four farms), was employed in relation to cheese making (Figure 2), alleviating concerns regarding synthetic chemical residues [34]. However, some farmers did not use it due to a lack of consensus on the efficacy of such treatments. The published studies also have limited scientific quality regarding CAVM, with a high or moderate risk of bias [37]. Two farmers’ utilisation of tree bark was likely associated with its use in combating gastrointestinal parasites in goats. Quebracho, a commercially available extract of the bark of a tropical tree (*Schinopsis* sp.), has demonstrated positive outcomes in tests [34]. Commercial nutraceuticals, comprising primarily plant-derived ingredients, are incorporated into phytotherapy. As they are not classified as drugs, there is no requirement for efficacy results, meaning that farmers must rely on their own assessment of these products. A plant that is to be included in a veterinary drug must comply with maximum residue limit regulations. More than 100 plants are listed as permitted substances [34], but the majority are only for homeopathic use, with only a few essential oils included. This may be a contributing factor to one farmer’s (F10) decision to abstain from using essential oils due to potential side effects and legal considerations. The distinction between organic and conventional farmers was less pronounced than in a study on meat sheep farming in central France [36]. It is notable that the terms “disease” and “phytotherapy” were more frequently mentioned among organic farmers, reflecting their tendency to adhere to CAVM-based disease treatment regulations. However, the study also found that some conventional farmers still preferred to use drugs, both organic and conventional, to treat diseases [3]. This preference was observed on one organic (F5) and one conventional farm (F4). This suggests that the use of CAVM, which was originally restricted to organic farmers, has now spread to conventional farming. The multivariate analysis of speech revealed that problems differed between organic and conventional farms (Figure 2). For organic farms, the main problems were the influence of seasons and milk production; and secondarily, paratuberculosis, parasitism, mud in the pasture, and the necessity to change. In contrast, conventional farms primarily highlighted challenges related to the intensity of work, cheese production, weaning periods, batch composition, goat health and diagnosis, silage and clay use, and stress at work. Organic farmers prioritised environmental concerns, including mud and parasitic infection in pastures, while conventional farmers focused on technical issues related to their work. Despite these differences in perspective, there was no significant difference in their reliance on CAVM. Despite utilising complementary medicine, farmers maintained regular contact with private veterinarians and belonged to a sanitary defence group comprising 8 of the 10 farms studied. Veterinarians were regarded as a reliable source of information on animal health, but their fees were a significant cost for farmers. One farmer expressed concerns about their limited knowledge of CAVM. This can be addressed in the future as there is now teaching on phytotherapy available in France [14]. Another farmer identified a lack of interest in dairy goats among private veterinarians as the reason for minimal interaction. There is a demand from farmers for an evolution in the role of veterinarians, like the changes seen in human medicine, where patients (in this case, farmers) transition from passive [38] to active participants [39] in health matters. It is expected that CAVM will be progressively more used in production animals, as evidenced in sport and companion animals [40].

## 5. Conclusions

Our study was based on the experiences of dairy goat farmers, who may not always reflect the actual practices related to CAVM. However, the study revealed that farmers already employ various CAVMs, and their eventual reluctance is driven by a lack of knowledge regarding the efficacy of these products. This underscores the necessity for enhanced evaluation of CAVMs’ efficacy and increased knowledge of their potential by veterinarians. These findings emerged from a local dairy goat production system using pastures, focused on milk and cheese production, distributed across a regional scale. Future research should quantify CAVM efficacy and expand to indoor systems.

## Figures and Tables

**Table 1:** Description of the 10 dairy goat farms studied in centre-west France.

Farm	Gender of Owner(s): Man (M) or Woman (W)	Education: Agricultural Diploma (A), Others (O), None *	Area of the Farm in ha	Number of Goats	Number of Working Units **	Cheese Making (1) or Not (0)	Organic (1) or Not (0)	CAVM ***
F1	M, W	A	42	100	3.5	1	1	Phytotherapy
F2	M	A	80	130	1.5	0	0	Phytotherapy
F3	M, W	None	80	125	3.5	1	0	Phytotherapy
F4	M, W	A	82	210	3	0	0	Phytotherapy, Homeopathy, Clay.
F5	M, W	A	160	400	10	1	1	Phytotherapy, Aromatherapy, Homeopathy, Tree bark.
F6	F, F	O, O	18	80	3	1	0	Phytotherapy Aromatherapy, Homeopathy, Clay, Vinegar.
F7	M	A	90	120	2.5	1	0	None
F8	M, W	A	40	260	2	0	0	Phytotherapy, Aromatherapy, Homeopathy.
F9	M	A	60	80	1	0	1	Phytotherapy
F10	M, W, W	A		140	3	1	0	Phytotherapy, Homeopathy, Osteopathy, Tree bark

* A: second-year university level in husbandry, O: third-year university level in biology or trade; ** full-time worker; *** complementary and alternative veterinary medicine.

**Table 2:** Occurrence of words in interviews with 10 dairy goat farmers from Touraine (centre-west France).

Characteristics	Factor	Number of Occurrences
Temporality	Year	55
	Time	16
Animals	Herd	24
	Horns	7
	Ear size	7
	Artificial insemination	5
Farm organisation	Work	34
	Organic	28
	Pasture	19
	Cereal	17
	Hectare	14
	Feed	9
Production	Milk	38
	Cheese	30
	Client and selling	24
Health	Treatment	37
	Veterinarian	27
	Mastitis	14
	Disease	13
	Foot bath	7
	Technician	7
	Parasitism	6
	Faecal egg count	6
	Disinfection	5
	Acidosis	4
	Paratuberculosis	4
Complementary medicine	Phytotherapy	13
	Aromatherapy	7
	Homeopathy	7
	Clay	6

## Data Availability

Already available in the article.

## Supplementary Materials

The following supporting information can be downloaded at: https://www.mdpi.com/article/10.3390/ani15111627/s1, Dataset S1: Guide for farmers’ interviews on their health practices and views.
