# Supplementary material for: Autonomous Farmers Use of Complementary and Alternative Veterinary Medicines in Pasture-Based Dairy Goat Systems
Source: Animals (Basel). 2025 May 31;15(11):1627. doi: 10.3390/ani15111627 (PMC12153671; doi:10.3390/ani15111627)
Supplement: Supplementary file 1 [file animals-15-01627-s001.zip › animals-3620658-supplementary.pdf]

Data set S1: Guide for farmer's interviews on their health practices and views.

1- Personal and professional background

History of the breeder and his profession(s)

How did you learn the trade? What have you learned since setting up that you didn't know before?

Can you describe the problems you faced in your early years?

How has your profession and your farm evolved? And around you?

What are the difficulties of being a dairy goat farmer?

(option for organic farming?)

2- The goat farming profession

What are the differences between goat farmers?

Several professions: which ones?

Diversification in the profession: cheese processing - other, sales, etc.

Organic/non-organic: what are the differences in the way the profession is conceived and practised?

What are the boundaries? How do they evolve? How do they change?

3 Work on the farm and its organisation

The current technical characteristics of the farm: number of animals, surface area, food self-sufficiency, processing, sales, etc.

How is your farm organised? How did you arrive at this organisation? Who does what?

Can you describe your work last week?

4 What collective activities?

Technical exchanges within the farm

Outside the farm: CETA, CIVAM, CUMA (agricultural machines), GAB (organic agriculture), neighbours, technicians, vets, GDS (Sanitary groups), etc.?

Who are your closest colleagues? Do you see each other often, what do you do together?

With whom do you discuss your work, are you in technical discussion groups?

5 How do you judge your work in goat farming?

What is a good goat farm? What are beautiful goats?

What is a good goat farmer's job? And the dirty work?

6 What is your health strategy and practice on your farm?

What is care work?

How do you monitor health?

Prevention: introductions, quarantine, genetics, rotational grazing, etc.

How does the problem of care arise on your farm?

What makes your farm different from others in terms of health?

The biggest health problems you've had, how did you solve them? Who did you rely on: colleagues, technicians (which ones), vet, etc. What was missing at the time; what posed a problem?

From a health point of view, what is the day-to-day work?

At this level, what do you consider to be doing your job well, because you have the time?

What do you consider to be doing a dirty job, because you're late?

What is a normal health situation in your farm?

How do genetics influence the health situation?

What is a hardy animal?

7 How do health problems manifest themselves?

What are the first indicators?

Do you have ‘sentinel’ animals?

Are there any risk situations on your farm?

What are the health histories on your farm: herd, flock, individual animals?

Do you use the expression ‘cruising herd’?

What is a health crisis: what are the thresholds, the transition from an individual to a batch, an epidemic, etc.?

What does it mean to work in a crisis? What happens, who do you see, who can you count on?

What impact do crisis situations have on the job: experiences, relationships with colleagues, etc.?

8 Health treatments

Which individual treatments/which batch treatments?

Synthetic allopathic treatments?

What phytopharmaceutical products do you use?

Phytotherapy, essential oils, etc.?

Why did you choose them: how, with whose help, and why did you choose them afterwards?

How do you treat the animals: injections, drinking, fogging, incorporating feed, manual applications?

Use of foot baths?

Do you disinfect your buildings; who does this work?

How do you manage your milking parlour?

9 How did you build up your sanitation experience, on what occasions, with whose help?

Which colleagues helped you and how?

Which technicians?

Which vets?

How do the animals help you identify their health problems?

How does the herd help you identify sick animals?

How does the sick animal help you spot it?

How do the animals contribute to their care (by calling on the breeder)?

How do the animals contribute to their care by self-medicating?

10 How do you write your working methods?

Do you have specifications?

How is this a constraint and how can it help with health?

How do you manage your breeding data: notes in a notebook, computerised, do you use technical and economic management tools?

11 How are the ways of working in goat farming and the profession changes?
